# How nurses support self-management of hospitalized patients through verbal communication: a qualitative study

**DOI:** 10.1186/s12912-022-01099-3

**Published:** 2022-11-28

**Authors:** Caroline E. M. Otter, Joost C. Keers, Celeste Reker, Jakobus Smit, Lisette Schoonhoven, Janneke M. de Man-van Ginkel

**Affiliations:** 1 Martini Hospital, Van Swietenlaan 1, Groningen, 9728 NT The Netherlands; 2 University of Applied Sciences Utrecht, Heidelberglaan 7, Utrecht, 3584 CS The Netherlands; 3 Julius Centre for Health Sciences and Primary Care, Nursing Science, University Medical Centre Utrecht, University Utrecht, HP Str. 6.131, Heidelberglaan 100, Utrecht, 3584 CX The Netherlands; 4 School of Health Sciences, Faculty of Environmental and Life Sciences, University of Southampton, University Road, Southampton, S017 1BJ UK; 5 Academic Nursing, Department of Gerontology and Geriatrics, Leiden University Medical Center, Albinusdreef 2, Leiden, 2333 ZA The Netherlands

**Keywords:** Self-management, Self-management support, Self-care, Hospital, Hospitalized adolescent, Verbal communication, Qualitative study, Observation

## Abstract

**Background:**

Patients’ self-management of the implications of their disease(s) is becoming increasingly important. Research shows that hospitalization disrupts established self-management routines. Nurses can play an important role in supporting patients’ self-management. The aim of this study is to describe how nurses support the self-management of hospitalized patients through verbal communication during routine nursing care.

**Methods:**

A qualitative descriptive study, using overt, non-participant observations was conducted on three wards of a general teaching hospital in the Netherlands. A total of 215 hours of nursing work during 49 shifts was observed. Data was analyzed using thematic analysis based on the six phases of Braun and Clarke.

**Results:**

Our observations showed that nurses discuss patients’ self-management mainly in short conversations during the care provision. Nurses ask patients about their self-management at home and stimulate patients to express their opinions and to be involved in the care process. Three themes reflect how nurses support self-management: ‘Discussing patient’s self-management’, ‘Enhancing patient’s involvement in care’ and ‘Focusing on patient’s perspective’.

**Conclusion:**

Hospital nurses have methods to support hospitalized patients’ self-management but it does not seem to be an integral part of daily practice. Given current developments in healthcare, it is reasonable to argue that self-management should be given greater emphasis within the hospital setting, requiring a collaborative approach with patients and other healthcare professionals across the care continuum.

**Supplementary Information:**

The online version contains supplementary material available at 10.1186/s12912-022-01099-3.

## Background

Healthcare systems in Western societies are changing from paternalistic systems toward systems that stimulate increasing active involvement of patients [1, 2]. This is especially evident in the care for people with chronic conditions at home [3, 4], but also relevant for people with non-chronic diseases [4] because they also need to self-manage the implications of their disease(s).

Self-management is usually considered as a subset of self-care and is focused on managing the consequences of health conditions [4, 5]. Self-management refers to the active participation of patients in their treatment [3, 4, 6] and include self-monitoring, symptom management and the management of functional, emotional, psychosocial and physical consequences of health conditions [4]. In this context ‘self’ is not understood literally as it also includes collaboration with family, community, and healthcare professionals [4, 5].

Self-management implies a participative collaboration with care providers [5, 6]. Both patients and caregivers are responsible, but the ultimate responsibility rest with the patients [3, 5]. They need to take part actively in the care process, and bear responsibility for the care process [6]. Health care professionals can support patients by working with patients in partnership, and by promoting patient activation, education and empowerment [5, 7], with the aim to encourage patients to use their own skills, information and professional services to take effective control of their life [5]. Interventions directed towards self-management of patients with chronic conditions are effective on clinical outcomes, self-management behavior, quality of life and reduced healthcare utilization [8–10], although reported effects are sometimes inconclusive [10]. It is clear that interventions have to be tailored to individual patients [9].

Research shows that hospitalization often disrupts established self-management routines [11]. Patients manage their own care at home, at hospital admission they switch to being a passive consumer and at discharge they have to resume self-management [11, 12]. While admitted to a hospital, most patients wish to manage their illness and situation as autonomously as possible and prefer to be actively involved in the care process [11, 13]. However, research found that patients often experience a lack of autonomy and involvement [13]. Patients often leave the hospital with inadequately preparation for self-management [12–14]. Challenges patients experience after a hospital admission are related to three areas: knowledge, resources and self-efficacy [12].

It can be argued that self-management should be supported during hospital stay, in order to maintain as much continuity in patients’ self-management as possible and to prepare the transition from hospital care to self-management after hospitalization [12, 14].

This is relevant for all patients, regardless of the reason for hospitalization. So far, most research has focused on support the self-management of community-dwelling patients with chronic diseases [3, 8]. In a hospital setting it is not desirable to distinguish between patients’ groups as it can be argued that all patients need self-management support. Making a distinction between patients with chronic and acute diseases would also be difficult, as transitions in disease states from acute to chronic occur [15]. Also, a lot of hospitalized patients have one or more chronic diseases [16], which may be the reason for the admission or not. Thus, regardless of the reason for hospitalization, patients must manage the consequences of their health problems.

Nurses can play an important role in supporting patients’ self-management [10, 17]. It is unclear how nurses support patients’ self-management while hospitalized, both with regard to maintaining continuity in patients’ self-management, as well as preparing patients to perform new self-management skills at home post discharge.

Communication is a core component of nursing [13, 18] intended to influence the patients’ health status or state of wellbeing [19]. Research has shown that supportive communication with patients can reduce uncertainty, enhance their engagement in decision-making, improve adherence to treatment plans, increase social support and encourage effective use of health care facilities [20].

## Methods

### Aim

This study aims to describe how nurses support the self-management of hospitalized patients through verbal communication during routine nursing care.

### Design

A qualitative descriptive study using overt, non-participant observation [21] and thematic analysis [22] was conducted to explore how nurses support inpatients self-management through communication. An overt non-participant observation means observing informed participants without participating in the observed activities [21].

### Setting and sample

The study took place at a general teaching hospital in the Netherlands. To get a broad picture of nursing care in a hospital with regard to self-management support, we chose to observe nurses providing direct care to hospitalized patients in three wards, a Medical, Surgical, and Dialysis ward. Nurses were asked to participate by their ward manager and informed verbally and in writing. They were told that the communication between nurse and patient would be observed. The ward managers recruited a diverse group of nurses, based on age, gender, educational level and years of experience. Nurses participated voluntarily and could refuse at any time. All participating nurses gave informed consent. Patients were asked permission for the observer being present during care to observe the nurse.

### Data collection

The observations were conducted by six student nurses (last year of training for bachelor in nursing degree) who have signed up for this graduation research. The students were specifically trained in non-participant observation and qualitative research and did not work on the participating wards before. The observations took place during day- and evening shifts, for 4–6 hours at a time, between April 6, 2018 and May 17, 2018.

To minimize the impact on the normal care situation, the observers looked like other student nurses and wore a uniform [21]. They did not participate in the nursing care provision. Each observer individually followed one nurse at a time. Communication with severely ill, delirious and/or palliative patients (based on participants’ clinical judgement) were not observed. When a patient’s bed curtains were closed, for example during personal care and treatments, the observer stayed outside. The observations lasted at least 4 h at a time to allow the nurse to get used to the observations. A maximum of 6 h’ observation time was agreed to ensure that observers remain concentrated during the observation.

#### Format for making field notes

A format for making field notes was developed by the chief investigator (CO), a female non-practicing nurse, employed at the hospital as a nursing researcher, not working in one of the participating nursing wards. This format includes sections regarding: 1) ward, date, observation start and end time, observer (number) and participating nurse (number), 2) nurses’ opinion about the workload during the shift (in normal or deviant), including motivation, 3) personal reflections of the observer during and after the observations (field diary), and 4) the communication, literally everything that was said, and the context (place, who is present, etc.). This format was pilot tested and discussed by the chief investigator (CO) and the observers [21].

Information about the age, gender, educational level and ward of the participating nurses were recorded to evaluate diversity in the sample. Anonymity of nurses was guaranteed by giving a number to each nurse. No patients’ characteristics were obtained.

#### The concept of self-management

In order to ensure a shared understanding of self-management we used the definition of self-management from the Dutch general nursing competency framework, which is based on the definition suggested by Barlow: “Self-management is the individual’s ability to prevent health problems wherever possible, and, when these still occur: to handle the symptoms, treatment, physical, psychological and social consequences of the health problems and the required lifestyle changes. This allows one to monitor and respond to his/her own state of health in a way that contributes to a satisfying quality of live” [1, 23]. There is no generally accepted description of how patients’ self-management during hospitalization is manifested in the daily (nursing) care. In this study, self-management during hospitalization was operationalized as: collaborating with the nursing staff and having a proactive role and control over personal care [24].

### Data analysis

Thematic analysis was conducted based on the six phases of Braun and Clarke [22], using Atlas-ti (version 8.0). Two members of the research team, namely CO and CR (a female transmural care consultant, MSc Sociology, working at the hospital but not in one of the participating wards), started the process by reading part of the transcribed material to obtain a broad overview of the content. In phase 2 both researchers (CO, CR) independently coded the same 12 documents inductively to generate initial codes. When searching for initial codes, the research question was kept in mind, but codes were primarily data driven. The initial codes found (*n* = 51) were discussed to establish consensus and then placed in a codebook. All documents were subsequently analyzed independently by the researchers, using the codebook. Two new codes were added during analysis. Data saturation was achieved, since the final documents analyzed did not present any new codes. In the following phase the initial codes were further analyzed through a careful exploration and study of all citations associated with the code. Some codes were merged, other codes were broken down further, which ultimately resulted in 65 codes. Subsequently, all codes were categorized into themes. An example of the analysis process is provided in Table 1. In phase 4 themes and categories were reassessed for overlap and the entire dataset was re-read to confirm that the themes fit in the data set and to code any additional data within the themes that has been missed in earlier coding. Each theme was clearly defined in a few sentences. In the last phases each theme and the corresponding sub-themes were named and illustrated with quotes. Both names and quotes were translated into English by one member of the research group (CO). This translation was verified by all members of the research group, including a native English speaker (JS). Finally, the data analysis was thoroughly discussed within the research team to reach consensus.

**Table 1:** Example of the analysis process regarding one theme

Quote (example)	Code	Subcategory	Category	Theme
Nurse: ‘Do you feel like eating?’	Asks wishes/opinion food/drink	Asks preferences and wishes	Asking patient’s opinion	Focusing on patient’s perspective
Nurse: ‘Would you like to sit on the bedside for breakfast?’	Asks wishes/opinion ADL/mobilizing			
Nurse: ‘What makes you so nauseous? Was it the Naproxen?’	Asks wishes/opinion medication			
Nurse: ‘Do you want to see the wound?’	Asks wishes/opinion care (involvement)			
Nurse: ‘Do I first have to flush the infusion?	Asks how action is done	Asks knowledge of (planned) care		
Nurse: ‘Do you want your blood sugar tested now?’- Patient: ‘It may also be done later during dialysis.’- Nurse: ‘When do they normally test?’	Asks for agreements made			
Nurse: ‘Do you want to have the Fraxiparine in the leg or in the abdomen?’	Offers a choice	Make a proposal or give a choice		
Nurse: ‘Hello, is it okay if I help you wash yourself now?’	Offers help			
Nurse: ‘Today 2600 cc fluid removal. What do you think of that?’	Makes a proposal			
Nurse: ‘Hi, I want to give you the Fraxiparine.’- Patient: ‘That’s okay.’	Names what he/she will do	Asks permission		
Nurse: ‘Hi, can I flush the tube?’	Asks permission to do something			
Patient: ‘I understood that I had to stop taking that one pill 7 days before the operation, but I got it this morning’. Nurse: ‘How nice of you to bring this up. Thanks for thinking along’.	Express appreciation for patient’s help or initiative	Accept patient’s initiative	Acknowledge patient’s initiative	
Patient: ‘My husband and son are coming soon, they want to take me in the wheelchair, so that I can get away for a while’. Nurse: ‘Okay’.	Confirm patient’s suggestion			
Patient: ‘Well, I prefer it (medication) in the evening, because otherwise I get so restless.’ Nurse: ‘Oh, that is good to know, I’ll have it changed.	Honor patient’s proposal or choice			
Nurse: ‘Today 2200 cc fluid removal.’- Patient: ‘Yes that’s fine, more is also okay. I will note 30 minutes in advance if I get cramps.’- Nurse: ‘But your blood pressure is low.’- Patient: Oh, then rather don’t do it, no.’- Nurse: ‘I will set it to 2200 cc.’- Patient: ‘That is okay.’	Offers a choice	Discuss patient’s initiative		

### Trustworthiness

Several strategies were applied to enhance trustworthiness. To enhance credibility we used multiple data sources in time (different times of the day), space (different wards) and persons (different nurses, different observers) and investigator triangulation (two researchers to make coding, analysis and interpretations decisions (CO and CR)) [25]. In addition, the observers were present on the wards for several weeks, to build trust and get acquainted with the context [25]. Furthermore, we provided information on the data analysis to illustrate how abstractions are made and gave representative quotations from the transcribed observations, which facilitates judging credibility [26]. To increase reliability we discussed researchers’ decisions and results within the research team and described the research steps of this study [25, 26]. The setting and the demographic characteristics of participating nurses were described to enable readers to put the findings in context and judge transferability to their own practice [25, 26].

## Findings

The sample consisted of 49 registered nurses, including male (*n* = 4) and female (*n* = 45) nurses with different educational levels (associate degree (*n* = 32); bachelor’s degree (*n* = 17), a varying amount of work experiences (mean 15.4 years; SD 13.8); and a mean age of 39 years (SD 14.7). A total of 215 hours of nursing work during 49 shifts was observed. Some observations lasted under 4 hours, mainly because a nurse’s shift ended earlier than planned. See Table 2 for an overview of the number of observations per type of ward.

**Table 2:** Overview of observations, per ward and per shift

Ward	Shift	Hours
Medical	6 morning	27
	6 afternoon	21
	7 evening	36
Surgical	5 morning	22
	2 afternoon	5
	5 evening	29
Dialysis	6 morning	39
	6 afternoon	18
	6 evening	18
**Total**		**215**

According to participating nurses most of the working days were normal. Sixteen observed shifts were judged to be deviant; more quit then normal [7], or busier than normal [9]. Personal reflections of the observers were mainly about the things that stood out in relation to self-management, such as more or less conversation with patients, or about being disturbed by others during the observation. Almost all communication consisted of short talks during nursing activities, often as a one-way transfer of information from nurse to patient or as a question from nurse to patient.

**Fig. 1:**
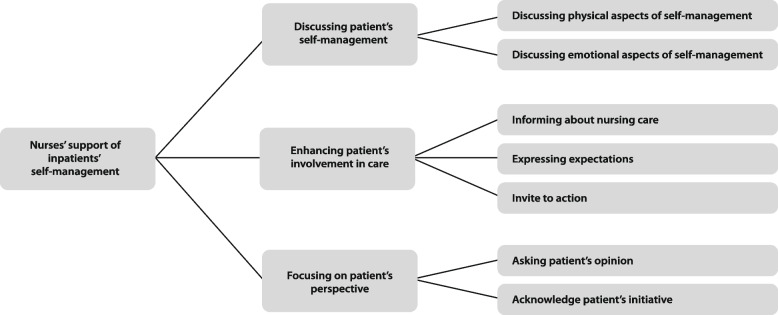
Themes and subthemes reflecting nurses’ support of inpatients’ self-management

The thematic analysis revealed three main themes and seven subthemes that reflect how nurses support inpatients’ self-management (See Fig. 1). In the following sections each of the main themes and their sub-themes are presented.

### Theme 1: discussing patient’s self-management

The data revealed that nurses pay attention to the way in which patients deal with their health problems. Two sub-themes in discussing the self-management emerged from the data, namely, discussing the management of physical consequences, and discussing the management of emotional aspects of the health condition(s) with the patients.

#### Discussing physical aspects of self-management

Nurses raised the issue of patients’ self-management by asking questions about the home situation and specifically about the way patients deal with health-related issues at home. This took place during some case history interview at admission and in short talks during the care provision. Questions during the case history interview were mostly aimed at screening for certain risks, such as the risk of falling or the risk of malnutrition. Questions were also asked about a prescribed diet, fluid restriction and medication use at home. Questions usually focused on factual information. Sometimes the nurse discussed the way the patient deals with self-monitoring at home, as is illustrated by this quote:

Nurse: ‘How often do you test (your blood sugar level) at home?’- Patient: ‘If it doesn’t feel right, I’ll check.’- Nurse: ‘With what result?’- Patient: ‘Good’ - Nurse: (Laughs) - Patient: ‘But sometimes it’s not good, therefore I always have dextrose with me.’ (Dialysis ward).

In a few situations nurses discussed patients’ self-management after discharge. For example, patients in the dialysis ward were encouraged to adhere to their regimen, such as fluid restrictions. In addition, patients were informed about medication use or were prepared for performing wound care independently at home, as one of the nurses demonstrated:

Nurse: ‘And did we mention you should rinse the wound after every bowel movement? And that a new bandage has to be put on’ – Patient: ‘No, not yet, but that makes sense. Otherwise it’s such a dirty bandage (laughs)’ – Nurse: ‘And after 24 hours the bandage has to be removed, unless you had a bowel movement’ - Patient: ‘Oh, yes, that is fine’ - Nurse: ‘It’s all written down in this letter, you can take it with you.’ (Surgical ward).

#### Discussing emotional aspects of self-management

Nurses asked patients about feelings related to their health situation, for example regarding a planned operation, the patient’s physical condition, or having to be on dialysis for years. The nurse showed understanding for the patient’s situation and sometimes mentioned possible solutions or motivations, as this nurse demonstrated:

Nurse: ‘How long have you been on dialysis?’ – Patient: ‘For eleven years.’ – Nurse: ‘Ever regretted it?’- Patient: ‘Regret, regret … ’. – Nurse: ‘If you want to carry on, you don’t really have a choice, huh.’- Patient: ‘No.’ (Dialysis ward).

Some patients shared their personal concerns, anxieties or fears. These concerns were generally focused on the patient’s own physical situation, for instance on having to mobilize again, or about whether the right care is provided in the right way. One patient mentioned being concerned about the future, possibly ending up in a nursing home. Nurses mostly responded to patients’ concern by reassuring the patient and by showing understanding. This was usually followed by providing some information, making a proposal or offering concrete help.

Patient: ‘ … .and last night something went wrong with the blood sugars too. So I need to be checked more often.’ – Nurse: ‘My colleague told me, we’ll keep a close eye on you today.’- Patient: ‘But if I don’t feel well, there must be someone.’- Nurse: ‘Yes, I’m nearby.’ (Medical ward).

### Theme 2: enhancing patient’s involvement in care

Data analysis revealed that nurses also support self-management by stimulating the patient’s involvement in nursing care. This is done in three ways namely, by giving information about the nursing care, by clearly indicating expectations towards the patient, and by inviting the patient to take an active role in personal care.

#### Informing about nursing care

Nurses provided information to the patient about the content and the planning of the nursing care and the motivation for these activities. Almost all nurses continuously specified what they were doing and what they plan to do next. Usually this information was general, brief, and given in combination with the performance of a nursing procedure. In some situations, nurses shared their considerations and observations with the patient, for example:

‘I’ve been thinking that maybe the infusion can be removed. You urinate well and you drink well. Only you need to eat a little better.’ (Surgical ward).

In addition, nurses gave information about the results of vital signs, medication, the dialysis, mobilizing and about aspects of daily living. These are occasionally combined with some advice. Nurses provided this information based on their own initiative or in response to patient’s questions. One nurse, for example, emphasized the importance of eating when blood sugar is low.

‘You still have a sandwich for later and you did have low blood sugar, so it’s wise to eat the sandwich.’ (Surgical ward).

In another example a nurse gave information to the patient about symptoms that he could monitor himself:

Nurse: ‘Do you know how to notice when you’re not doing well?’- Patient: ‘No.’- Nurse: ‘I’ll tell you. You may become dizzy, have blurred vision or you’ll sweat more. Or you may experience pain or have cramps. Basically anything that is not normal.’- Patient: ‘Okay, then I’ll call.’ (Dialysis ward).

#### Expressing expectations

Nurses also encouraged the patients to take an active role in personal care by expressing their expectations towards patients and by naming activities that the patient can perform on their own, mostly activities of daily living and using medication, as is illustrated by this quote:

‘I’ll put the bag (with medication) here, so you can decide for yourself when to use it.’ (Medical ward).

Nurses regularly indicated that they expect the patient to ask for help when needed. In addition of this frequently stated general question, more specific expectations towards the patient were expressed. Nurses expected patients to report when physical complaints worsen or when particular situation or symptom occurs. Some nurses asked patients to remind them to perform planned care:

‘Oh and before you eat I have to measure your (blood) sugar, please help me remember, will you let me know?’ (Dialysis ward).

#### Invite to action

In order to stimulate the patient’s involvement in the care process patients were also directly invited to participate in the provision of nursing care. This mainly took place with regard to the activities of daily living, such as bathing or changing patient’s physical position in bed.

On occasion nurses would ask the patient to play a role in performing a concrete nursing procedure. This was common in the dialysis ward and occurred incidentally in other nursing wards. In the dialysis ward almost all patients had a role in puncturing and removing the tubes, as demonstrated in this quote:

‘If you hold the dialysis tubes with your right hand, then you will be my assistant’ (Dialysis ward).

Sometimes the patient would be invited to self-manage medication intake. One example is this nurse discussing patients’ inhaler:

Nurse: ‘Do you have your own inhaler?’ – Patient: ‘Yes’ – Nurse: ‘And you use it yourself?’ – Patient: ‘Yes of course.’ (Surgical ward).

### Theme 3: focusing on patient’s perspective

This theme describes the communication in which nurses demonstrated how they took the patient’s perspective into account. Two sub-themes emerged from the analysis namely, asking the patient’s opinion and acknowledging the patient’s initiative.

#### Asking patient’s opinion

Patients were encouraged to indicate their thoughts about the nursing care. Nurses did this in several ways. First, nurses asked for patients’ preferences, especially regarding activities of daily living, or taking medication.

Nurse: ‘You’re still in the chair. Are you okay? Or do you want to go back to bed?’ (Surgical ward).

In some situations, the patient’s preference on other issues were asked, as the question below illustrates:

Nurse: ‘Do you want to see the wound?’- Patient: ‘No, not yet.’ (Surgical ward).

Secondly, nurses asked patients about the agreements made about the provision of nursing care. This mainly took place in the dialysis ward. In this ward patients do have a relatively large say in determining how nursing care is provided, for example with regard to the timing of activities:

Nurse: ‘Do you want your blood sugar tested now?’- Patient: ‘It may also be done later during dialysis.’- Nurse: ‘When do they normally test?’- Patient: ‘Usually before eating, but actually it has to be done one hour after eating and I have just eaten.’ (Dialysis ward).

Thirdly, nurses presented patients with a choice or with a concrete proposal regarding nursing care. These choices mainly related to minor decisions such as an injection in the abdomen or leg or whether an action would take place now or later. Nurses also made concrete proposals to patients, focusing on activities related to daily living or taking medication, and on a nursing procedure, such as how much fluid will be extracted during dialysis:

Nurse: ‘Today 2600cc fluid removal. What do you think of that?’- Patient: ‘Yes, that should work.’ (Dialysis ward).

Last, nurses asked permission from patients to perform a nursing action. This usually involved checking vital signs or conducting certain nursing interventions. This request for consent from patients was given explicitly, but also implicitly. The nurse indicated that she would like to perform an action, to which the patient indicated that this is approved, as this nurse showed:

Nurse: ‘Hi, I want to give you the fraxiparine.’- Patient: ‘That’s okay.’ (Medical ward).

#### Acknowledge patient’s initiative

Nurses also focused on the patients’ perspective by acknowledging initiatives taken by patients. Such initiatives include presenting specific requests, by asking questions, by giving instructions to the nurse, or simply by doing something themselves. These initiatives were aimed at activities related to daily living, the intake of medicine, the planning of care and certain nursing procedures, such as removing stitches.

Nurses responded positively to the patients’ initiative in two ways. In most cases nurses accepted the patient’s suggestion, agreed with the patient’s proposal and indicated that they valued the patient’s own initiative, as illustrated with this quote:

Patient: ‘I don’t have my medication.’ – Nurse: ‘We have it. I see it’s already written in here, and you’ll get the medication at 10 AM.’- Patient: ‘Well, I prefer it in the evening, because otherwise I get so restless.’- Nurse: ‘Oh, that is good to know, I’ll have it changed.’ (Surgical ward).

In some situations, the nurse discussed alternatives regarding patients’ proposal and they decided together what to do, for example regarding fluid removal:

Nurse: ‘Today 2200cc fluid removal.’- Patient: ‘Yes that’s fine, more is also okay. I will note 30 minutes in advance if I get cramps.’- Nurse: ‘But your blood pressure is low.’- Patient: Oh, then rather don’t do it, no.’- Nurse: ‘I will set it to 2200cc.’- Patient: ‘That is okay.’ (Dialysis ward).

## Discussion

The analysis revealed that nurses support self-management of hospitalized patients in a direct way, through ‘Discussing patient’s self-management’ and in indirect ways, by ‘Enhancing patient’s involvement in care’; and ‘Focusing on patient’s perspective’.

When nurses discuss patients’ self-management, they seem to have little attention for the patients’ self-management behavior before the hospital admission. Only a few case history interviews were observed, although the information from this interview is necessary for developing a personal nursing care plan. It is likely that interactions where information regarding behavior before admission was discussed took place in settings that were not encountered by observers and that this information may already have been included in the nurses’ documentation.

Patients’ self-management after discharge was not discussed with all patients. This is in line with previous studies, which indicated that teaching self-management skills is not part of hospital care [13, 14, 27]. It is important to prepare patients for self-management at home. Many patients have low health literacy and find it difficult to interpret and understand basic medical information and in translating this information into action [28, 29]. Nurses and other health care professionals often overestimate some patients’ health literacy [30], therefore it may be wise to assume that all patients may have difficulty understanding information and to create an environment where all patients can improve their understanding and basic self-management skills during hospitalization [14, 28].

Nurses also paid attention to patients’ self-management by indirect methods. We discovered two approaches: through involving the patient in the nursing care; and by paying attention to what the patient considers to be important. These approaches can be seen as strategies to stimulate individual patient participation, which can lead to greater patient empowerment and the improvement of patients’ self-management [2].

Self-management is daily work for the patient [3]. This does not stop when a patient is hospitalized. An admission is a great opportunity to give patients education in self-management skills [31]. It can be the start for enhancing skills needed for effective self-management, such as problem solving, decision making, self-monitoring and symptom management, and for developing a behavior change action plan [3, 8]. In addition, patients can be prepared prior to hospitalization to perform self-management before, during and after hospitalization [32–34].

As far as we know, the way in which nurses support self-management during hospitalization has not been studied before. Findings from a non-participant observation looking at the role of nurses in health promoting in the acute hospital setting showed that nurses conducted health education in a traditional way and that patient participation was limited to small personal aspects of care [35]. This was also the case in the current study, which shows that nurses encouraged patients to participate in their own care, mostly regarding the activities of daily living. Nurses see inpatients’ involvement in these activities as a starting point for performing more self-management tasks [36]. However, additional action has to be taken to maintain as much continuity in patients’ self-management as possible and to prepare the transition from hospital to home. During hospitalization, patients may also be involved in activities aimed at managing the impact of their condition, such as managing symptoms or preventing complications, which they also need to perform at home. This will help them to maintain self-confidence and allow them to develop new self-management skills while in the hospital [12].

We looked at the content of the communication exchanges between nurses and patients, with a focus on the role of nurses in this. As reported in other studies, communication often happens while performing other tasks [19, 37]. Nurses often communicate in a task-focused manner by focusing on physical care. Effective self-management support should also pay attention to patients’ emotional and psychosocial needs regarding the consequences of their condition(s) [4].

Nurses did stimulate some form of partnership in the care by asking patients’ view on nursing activities or asking for patients’ assistance with conducting nursing care. This took place in all wards, but most often in the dialysis ward. Dialysis patients had a relatively large input in determining how nursing care is provided, probably because they are familiar with the nursing staff and the nursing procedures because they are admitted several times a week to undergo dialysis. This can be regarded as inpatients’ self-management since the patient collaborated with nursing staff, was proactive and gave direction to, and had control over personal care [24]. In the other wards the decisions nurses handed over to the patient were limited to minor personal aspects of care. Some nurses explain the connection between vital signs and the nursing care, stimulating a patient’s understanding how these parameters can be influenced and in only a few situations the patient was prepared for self-monitoring symptoms or invited to take responsibility for using medication while hospitalized. Aforementioned can be regarded as examples of strengthening patients’ self-management skills during hospitalization.

Our findings indicate that nurse do have methods to support self-management of hospitalized patients, but they do not support all of the patients’ possible self-management needs and these methods do not appear to be used in all relevant patients’ encounters. Health care professionals seem not to be skilled to sufficiently perform self-management support [17, 36]. Traditionally, they are trained to take responsibility for patients’ acute health problems instead of engaging patients as partners in their care [17, 38]. Nurses find it difficult to release professional control and have little confidence in patients’ ability to manage their health well [17, 36, 39]. In addition, nurses experience differing expectations from patients, managers and colleagues regarding self-management support [36]. Most nurses working in the acute hospital care do not know what the patient needs for effective self-management and how to support the patient in this [36]. An unclear role definition can affect nurses’ responses to patients [40]. Therefore, in order to improve nurses’ support to hospitalized patients’ self-management, nurses need knowledge, skills, a clear policy and clarity about their role. Specialized nurses are often additionally trained to provide self-management education. However, supporting inpatients’ self-management requires adequate competences from all nurses and other health care providers [41]. Theory-driven training interventions, with time to practice, (video) feedback and follow-up seem to be the most effective to train nurses’ competences in self-management support [42].

In addition to training nurses’ competences, self-management support programs should include patient-centered elements, such as involving patients as partners, and organizational aspects, such as having a multidisciplinary team approach [41]. To ensure continuity in care, programs aimed at enhancing patients’ self-management are best developed across the patients’ entire care pathway. This requires a joint approach in which patients, home healthcare, primary care, hospital care and long-term care work together.

Observational research contributes to our understanding of current practices. This study provides an initial, general presentation of what nurses do to support inpatients’ self-management. Since there is little previous research in this area, we tried to obtain a broad overview of the practice. We have chosen to observe how nurses support the patient’s self-management during hospitalization and not to map whether nurses do this in all possible and appropriate situations. To develop the findings further, additional research is needed with focused and selective observations and discussing nurses’ perspectives on the meaning of what was observed, to enhance the understanding of nurses’ communication in support of inpatients self-management [43].

## Limitations

This study provides insight into nurses’ support of inpatients’ self-management in one hospital, which may limit the transferability of the findings to other settings. In addition, the use of observation as a method of data collection implies a danger that the act of observing may alter practice; the so-called ‘Hawthorne effect’ [21]. However, it is reasonable to assume that this effect was limited because participating nurses regularly supervise and train student nurses and thus are used to being observed by students while performing their duties.

Finally, observations were conducted by student nurses, which may cause bias as a result of inexperience despite the training they received. On the other hand, being a student nurse can provide an open, unbiased view.

## Conclusion

Considering current developments in health care and the changing view on health it can be argued that self-management needs to be emphasized more, also within a hospital setting. It appears that nurses pay attention to supporting hospitalized patients’ self-management in several ways, but this seems to be done ad hoc and does not focus on all patient’s possible self-management needs. Self-management support should be embedded in policy at organization and ward level. Interventions should be developed that support patients’ ability to manage their health condition across the care continuum.

## Supplementary Information

**Additional file 1.**

## Data Availability

All data used and analyzed during this study are in Dutch and available from the corresponding author on request.
